# Performance of Sequential Organ Failure Assessment and Simplified Acute Physiology Score II for Post-Cardiac Surgery Patients in Intensive Care Unit

**DOI:** 10.3389/fcvm.2021.774935

**Published:** 2021-12-06

**Authors:** Fei Xu, Weina Li, Cheng Zhang, Rong Cao

**Affiliations:** Department of Anesthesiology, Chengdu Women's and Children's Central Hospital, Chengdu, China

**Keywords:** post-cardiac surgery, SOFA, SAPS II, clinic outcome, intensive care unit

## Abstract

**Background:** The aim of this study is to assess the performance of Sequential Organ Failure Assessment (SOFA) score and Simplified Acute Physiology Score (SAPS II) on outcomes of patients with cardiac surgery and identify the cutoff values to provide a reference for early intervention.

**Methods:** All data were extracted from MIMIC-III (Medical Information Mart for Intensive Care-III) database. Cutoff values were calculated by the receiver-operating characteristic curve and Youden indexes. Patients were grouped, respectively, according to the cutoff values of SOFA and SAPS II. A non-adjusted model and adjusted model were established to evaluate the prediction of risk. Comparison of clinical efficacy between two scoring systems was made by decision curve analysis (DCA). The primary outcomes of this study were in-hospital mortality, 28-day mortality, 90-day mortality, and 1-year mortality after cardiac surgery. The secondary outcomes included length of hospital stay and intensive care unit (ICU) stay and the incidence of acute kidney injury (AKI) within 7 days after ICU admission.

**Results:** A total of 6,122 patients were collected and divided into the H-SOFA group (SOFA ≥ 7) and L-SOFA group (SOFA < 7) or H-SAPS II group (SAPS II ≥ 43) and L-SAPS II group (SAPS II < 43). In-hospital mortality, 28-day mortality, 90-day mortality, and 1-year mortality were higher, the length of hospital and ICU stay were longer in the H-SOFA group than in the L-SOFA group (*p* < 0.05), while the incidence of AKI was not significantly different. In-hospital mortality, 28-day mortality, 90-day mortality, 1-year mortality, and the incidence of AKI were all significantly higher in the H-SAPS II group than in the L-SAPS II group (*p* < 0.05). Hospital stay and ICU stay were longer in the H-SAPS II group than in the L-SAPS II group (*p* < 0.05). According to DCA, the SAPS II scoring system had more net benefits on assessing the long-term mortality compared with the SOFA scoring system.

**Conclusion:** Exceeding the cutoff values of SOFA and SAPS II scores could lead to increased mortality and extended length of ICU and hospital stay. The SAPS II scoring system had a better discriminative performance of 90-day mortality and 1-year mortality in post-cardiac surgery patients than the SOFA scoring system. Emphasizing the critical value of the scoring system is of significance for timely treatment.

## Introduction

Prognosis is a common challenge for patients after cardiac surgery. Although some progress has been achieved in the application of cardiac surgery procedures, the mortality rates after surgery remain high. Some scoring systems played an important role in the successful prediction of cardiac surgery-related mortality (1, 2). There was a preoperative risk stratification model, which had been widely accepted for mortality prediction of in-hospital mortality after cardiac surgery (3).

However, the scoring system focused on preoperative indicators without attention to intraoperative or postoperative conditions (4). The severity of surgical stress and inflammatory response to cardiopulmonary surgery could not be ignored (5) in cases that were associated with organ dysfunction and acute physiological changes. Previous studies reported that hyperlactatemia, bicarbonate, heart rate, and creatinine were essential for post-cardiac surgery patients as mortality-predictive variables (6–8). Recent studies had confirmed that the neutrophil/lymphocyte ratio of post 24 h after intensive care unit (ICU) admission is associated with postoperative mortality of cardiac surgery (9). Parameters and physiological indexes of post 24 h after ICU admission may provide a new way to improve prognosis early.

The Sequential Organ Failure Assessment (SOFA) score and Simplified Acute Physiology Score (SAPA II) were composed of organ function and biochemical indexes, which showed excellent predictive performance on many diseases in ICU (10, 11). There were few pieces of small sample literature that explored the role of SAPS II and SOFA scoring systems in predicting poor prognosis after cardiac surgery (12). The purpose of this study was to determine the critical values of the SAPS II and SOFA scoring system and to evaluate their performance in predicting the prognosis of patients undergoing cardiac surgery.

## Materials and Methods

### Sources of Data

All data in this study were retrospectively extracted from MIMIC-III (Medical Information Mart for Intensive Care-III) database (13, 14). This is a freely open database for the public, which includes more than 40,000 critically ill patients from Beth Israel Deaconess Medical Center (Boston, Massachusetts, United States) between 2001 and 2012. The application of the database was approved by the institutional review committee of Beth Israel Deaconess Medical Center and Beth Israel Deacons Medical Center (Approval Code 10323541). Patient-related information in the database was anonymous, and personal informed consent was abandoned in this study.

### Data Collection and Definitions

The structure query language (SQL) with code in MIMIC Code Repository (https://github.com/MIT-LCP/Mimic-Website) was used for extracting data. The whole variables involved basic characteristics (age, gender, body mass index), comorbidities (drug abuse, alcohol abuse, coagulopathy, liver disease, hypertension, hypothyroidism, congestive heart failure, diabetes, chronic lung disease, chronic kidney disease), laboratory tests (sodium, potassium, white blood cell counts, hemoglobin, platelet, lactate, creatinine, blood urea nitrogen, prothrombin time, international normalized ratio, glucose), and vital signs (heart rate, respiratory rate, body temperature, pulse oxygen saturation, diastolic pressure, systolic pressure, and mean arterial pressure). The SOFA score and SAPS II score were evaluated within the first 24 h after ICU admission. Variables were reported as the average value within 24 h admitted to ICU.

Patients with cardiac surgery whose diagnose code ranged from 33,010 to 37,799 were identified using current procedural terminology. Inclusion criteria were patients aged between 18 and 89 years, choosing the first hospitalization for analysis when admitted to hospital or ICU multiple times. Patients hospitalized in ICU <24 h were excluded. The primary outcomes were in-hospital mortality, 28-day mortality, 90-day mortality, and 1-year mortality after cardiac surgery. The secondary outcomes were the length of hospital stay, ICU stay, and the incidence of acute kidney injury (AKI) within 7 days after ICU admission. The diagnosis of AKI referenced the improving global outcomes guideline (15).

### Statistical Methods

Continuous variables of the study were non-normally distributed and reported as medians along with interquartile ranges (IQRs). Categorical variables were presented as numbers and percentages. Comparison of different groups was made using Kruskal–Wallis test or Mann–Whitney U-test for continuous variables, whereas chi-square or Fisher's exact tests were used for categorical variables. Non-adjusted models and adjusted models were established to investigate the association between scoring systems and outcomes in this study.

The cutoff values of SOFA and SAPS II scoring systems were obtained across receiver-operating characteristic (ROC) curve and Youden Indexes calculation. Based on different cutoff values, patients were divided into two groups. The clinical efficacy of scoring system models for poor outcomes was assessed by decision curve analysis (DCA), which was considered an appropriate method for estimating prognostic strategies (16). Kaplan–Meier curve was used to describe the difference in 1-year survival between different groups of the scoring system.

The SPSS software version 24.0 (IBM Corporation, Armonk, NY, United States) and the R software (version 4.0.3) were used for data processing, statistical analysis, and illustrations. Data missing 30% were removed and data missing <30% were processed by multiple imputations. Statistical differences were set at *p* < 0.05.

## Results

### Clinical Characteristics

**Figure 1:**
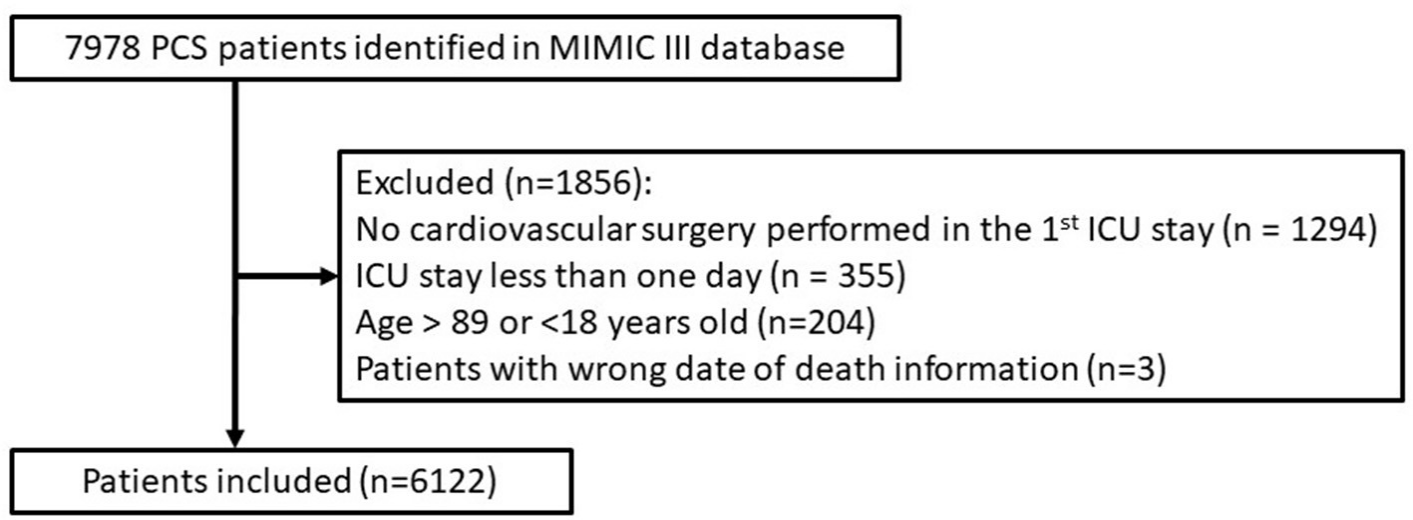
Flowchart of study cohort selection.

A total of 6,122 patients admitted to ICU after cardiac surgery were enrolled retrospectively. The procedures and standards for data selection are shown in Figure 1. The clinical basic characteristics of the populations are shown in Table 1.

**Table 1:** Baseline clinical characteristics between survivors and non-survivors at 1 year.

**Characteristics**	**Overall (*n* = 6,122)**	**Survivors (*n* = 4,516)**	**Non-survivors (*n* = 1,606)**	***p***
Age, median (IQR)	66 (55, 76)	65.0 (54.0, 74.0)	69.0 (58.0, 79.0)	<0.001
Male gender, *n* (%)	3,704 (60.503)	2,790 (61.780)	914 (56.912)	<0.001
BMI, median (IQR)	27.84 (24.39, 31.84)	28.04 (24.67, 32.04)	27.06 (23.55, 31.24)	<0.001
**Comorbidities**				
Drug abuse, *n* (%)	208 (3.398)	156 (3.454)	52 (3.238)	0.681
Alcohol abuse, *n* (%)	474 (7.743)	331 (7.329)	143 (8.904)	0.043
Coagulopathy, *n* (%)	1,153 (18.834)	661 (14.637)	492 (30.635)	<0.001
Congestive heart failure, *n* (%)	816 (13.329)	384 (8.503)	432 (26.899)	<0.001
Liver disease, *n* (%)	463 (7.563)	250 (5.536)	213 (13.263)	<0.001
Renal failure, *n* (%)	869 (14.195)	526 (11.647)	343 (21.357)	<0.001
hypothyroidism, *n* (%)	617 (10.078)	460 (10.186)	157 (9.776)	0.639
Diabetes, *n* (%)	1,448 (23.652)	1,080 (23.915)	368 (22.914)	0.418
Chronic pulmonary, *n* (%)	1,179 (19.258)	818 (18.113)	361 (22.478)	<0.001
hypertension, *n* (%)	758 (12.382)	481 (10.651)	277 (17.248)	<0.001
**Laboratory test**				
WBC mean, median (IQR)	11.93 (8.9, 15.55)	11.85 (9.13, 15.2)	12.3 (8.1, 16.74)	0.347
BUN mean, median (IQR)	18.33 (13.0, 29.4)	16.5 (12.5, 23.5)	28.5 (18.0, 47.5)	<0.001
Sodium mean, median (IQR)	138.0 (136.0, 140.2)	138.0 (136.2, 140.0)	138.4 (135.33, 141.5)	0.002
PT mean, median (IQR)	14.65 (13.6, 16.2)	14.47 (13.5, 15.7)	15.6 (13.95, 18.6)	<0.001
INR mean, median (IQR)	1.3 (1.2, 1.5)	1.3 (1.2, 1.4)	1.43 (1.2, 1.8)	<0.001
Potassium mean, median (IQR)	4.19 (3.87, 4.5)	4.2 (3.9, 4.49)	4.15 (3.8, 4.6)	0.095
Platelet mean, median (IQR)	175.33 (130.33, 233.33)	176.33 (136.0, 228.5)	172.0 (109.67, 253.5)	0.007
Lactate mean, median (IQR)	1.93 (1.4, 2.7)	1.9 (1.4, 2.6)	2.0 (1.4, 3.1)	<0.001
Hemoglobin mean, median (IQR)	10.15 (9.15, 11.4)	10.2 (9.18, 11.4)	10.0 (9.05, 11.3)	0.001
Glucose mean, median (IQR)	130.57 (116.0, 150.89)	129.67 (116.43, 146.5)	135.25 (114.4, 166.86)	<0.001
Creatinine mean, median (IQR)	0.95 (0.72, 1.37)	0.9 (0.7, 1.18)	1.27 (0.83, 2.24)	<0.001
PH mean, median (IQR)	7.38 (7.34, 7.41)	7.38 (7.34, 7.41)	7.37 (7.31, 7.42)	<0.001
**Vital sign**				
SpO_2_ mean, median (IQR)	97.77 (96.52, 98.81)	97.86 (96.72, 98.86)	97.47 (95.88, 98.7)	<0.001
BT mean, median (IQR)	36.81 (36.43, 37.24)	36.85 (36.47, 37.25)	36.74 (36.32, 37.21)	<0.001
Resp rate mean, median (IQR)	18.12 (16.08, 21.17)	17.7 (15.86, 20.25)	20.0 (17.19, 23.69)	<0.001
Mean bp mean, median (IQR)	75.11 (70.17, 81.36)	75.44 (70.79, 81.27)	74.02 (68.07, 81.73)	<0.001
Dias bp mean, median (IQR)	58.39 (53.15, 64.3)	58.64 (53.77, 64.37)	57.51 (51.26, 64.07)	<0.001
Sys bp mean, median (IQR)	112.86 (105.52, 122.26)	113.35 (106.45, 122.03)	111.22 (102.62, 123.08)	<0.001
Heartrate mean, median (IQR)	85.83 (77.53, 96.52)	84.77 (77.35, 94.74)	88.91 (78.05, 101.72)	<0.001
**Score system**				
SPAS II, median (IQR)	38 (30, 48)	35.0 (28.0, 44.0)	48.0 (38.0, 57.0)	<0.001
SOFA, median (IQR)	5 (3, 8)	5.0 (3.0, 7.0)	7.0 (4.0, 10.0)	<0.001
AKI 7-day, (%)	4,563 (74.534)	3,268 (72.365)	1,295 (80.635)	<0.001
Hospital stay ≥ 14 days, *n* (%)	2,092 (34.172)	1,359 (30.093)	733 (45.641)	<0.001
ICU stay ≥ 3 days, *n* (%)	3,422 (55.897)	2,265 (50.155)	1,157 (72.042)	<0.001
Survival time, median (IQR)	11.15 (6.26, 27.17)	9.89 (6.08, 19.68)	22.2 (8.08, 78.7)	<0.001

*BMI, body mass index; WBC, white blood cell count; BUN, Blood urea nitrogen; PT, prothrombin time; INR, international normalized ratio; SOFA, sequential organ failure assessment; SPASII, simplified acute physiology score; AKI, acute kidney injury; ICU, the intensive care unit; SpO_2_, pulse oxygen saturation; PH, potential of hydrogen; BT, body temperature; Data are represented as median (interquartile range) or n (%)*.

**Figure 2:**
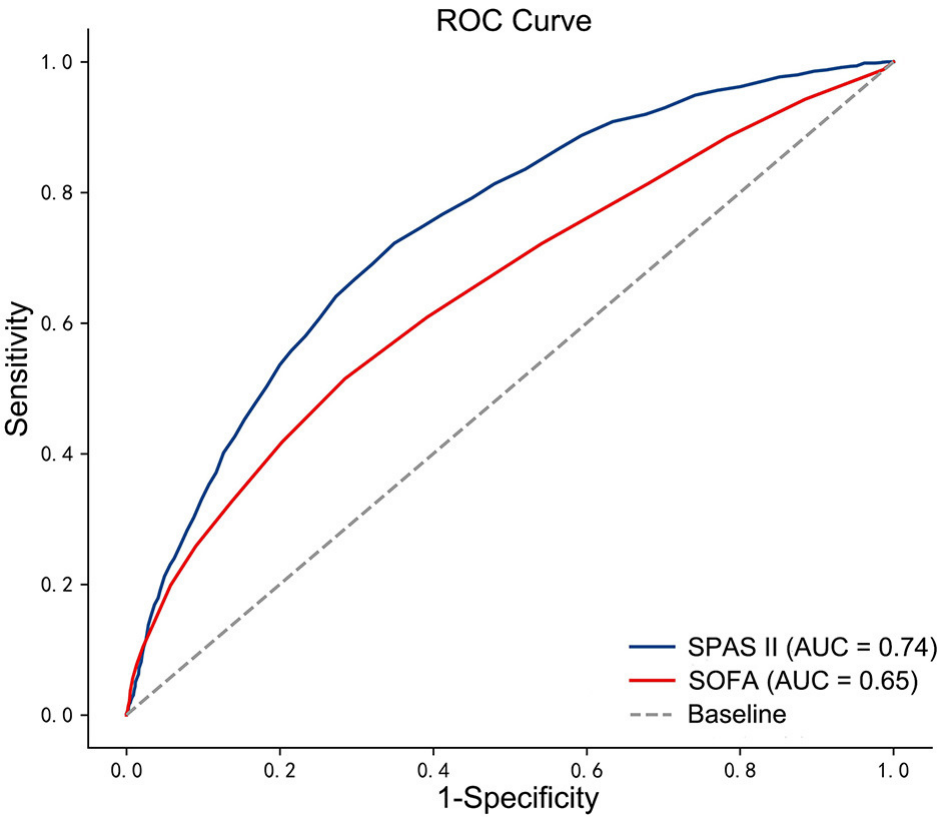
The ROC curves for SOFA score and SAPS II score with 1-year postoperative mortality. ROC, receiver-operating characteristic.

**Figure 3:**
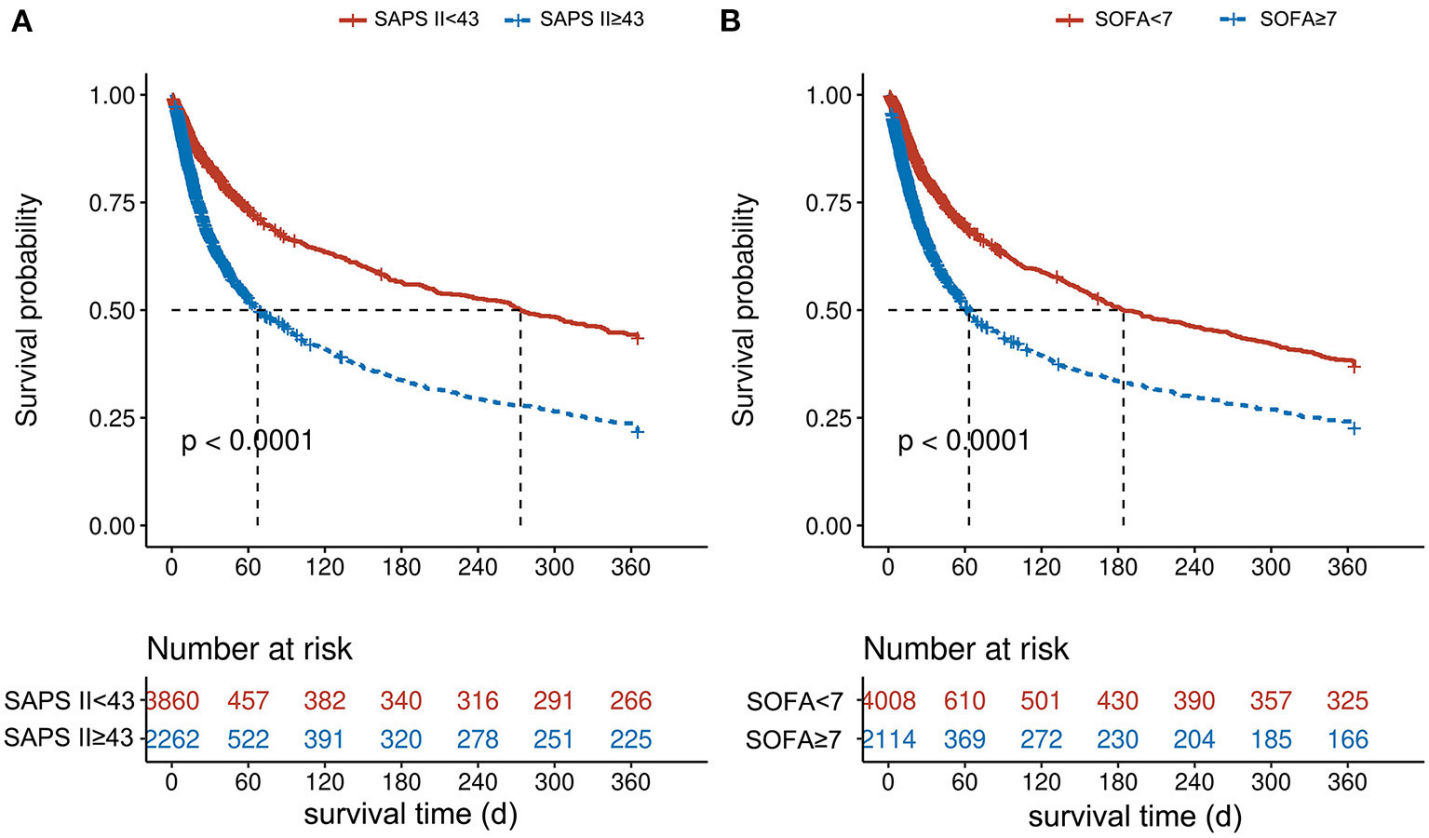
Kaplan–Meier curves of 1-year mortality by scoring system groups. **(A)** For the SAPS II score, while **(B)** is for the SOFA score.

The AUC of SOFA and SAPS II scoring systems with 1-year postoperation mortality was 0.649 (*p* < 0.001) and 0.724 (*p* < 0.001), respectively (Figure 2). The cutoff value of the SOFA scoring system was 7, whereas the critical value of the SAPS II scoring system was 43. In the SOFA scoring system, the patients were divided into high SOFA group (H-SOFA group, SOFA ≥ 7, *N* = 2,114) and low SOFA group (L-SOFA group, SOFA < 7, *N* = 4,008). In the SAPS II scoring system, patients were divided into the high SAPS II group (H-SAPS II group, SAPS II ≥ 43, *N* = 2,262) and low SAPS II group (L-SAPS II group, SAPS II < 43, *N* = 3,860). Kaplan–Meier survival curves of scoring system groups were statistically different (log-rank *p* < 0.001, Figure 3).

### Correlation Between SOFA Scoring System and Postoperative Outcomes

General comparative data of population are reported in Table 2 and further explored in Table 3. Both short-term and long-term mortality rates were statistically significant in the comparison between SOFA scoring system groups. In-hospital mortality [odds ratio (OR) 2.86, 95% confidence interval (CI) 2.471, 3.312; *p* < 0.001], 28-day mortality (OR 2.897, 95% CI 2.528,3.323; *p* < 0.001), 90-day mortality (OR 2.817, 95% CI 2.475,3.208; *p* < 0.001), 1-year mortality (OR 2.479, 95% CI 2.195, 2.8; *p* < 0.001) all increased in the H-SOFA group compared with the L-SOFA group. In addition, length of in-hospital stay (OR 1.744, 95% CI 1.557, 1.954; *p* < 0.001) and ICU stay (OR 2.444, 95% CI 2.18, 2.743; *p* < 0.001) was extended in the H-SOFA group. However, there were no significant relationships in the incidence of acute renal failure (AKI) within 7 days after ICU admission (*p* < 0.05) between the two groups.

**Table 2:** Basic characteristics of SOFA scoring system.

**Characteristics**	**Overall (*n* = 6,122)**	**SOFA <7 (*n* = 4,008)**	**SOFA≥7 (*n* = 2,114)**	***p***
SOFA, median (IQR)	5 (3, 8)	4.0 (2.0, 5.0)	9.0 (8.0, 11.0)	<0.001
Age, median (IQR)	66 (55, 76)	66.0 (55.0, 76.0)	66.0 (55.0, 76.0)	0.793
Male gender, *n* (%)	3,704 (60.503)	2,385 (59.506)	1,319 (62.394)	0.028
BMI, median (IQR)	27.84 (24.39, 31.84)	27.62 (24.22, 31.59)	28.25 (24.69, 32.28)	<0.001
**Comorbidities**				
Drug abuse, *n* (%)	208 (3.398)	126 (3.144)	82 (3.879)	0.131
Alcohol abuse, *n* (%)	474 (7.743)	253 (6.312)	221 (10.454)	<0.001
coagulopathy, *n* (%)	1,153 (18.834)	464 (11.577)	689 (32.592)	<0.001
Liver disease, *n* (%)	463 (7.563)	193 (4.815)	270 (12.772)	<0.001
Renal failure, *n* (%)	869 (14.195)	405 (10.105)	464 (21.949)	<0.001
hypothyroidism, *n* (%)	617 (10.078)	417 (10.404)	200 (9.461)	0.244
diabetes, *n* (%)	1,448 (23.652)	934 (23.303)	514 (24.314)	0.376
Chronic pulmonary, *n* (%)	1,179 (19.258)	775 (19.336)	404 (19.111)	0.831
hypertension, *n* (%)	758 (12.382)	373 (9.306)	385 (18.212)	<0.001
Congestive heart failure, *n* (%)	816 (13.329)	449 (11.203)	367 (17.360)	<0.001
**Laboratory test**				
WBC mean, median (IQR)	11.93 (8.9, 15.55)	11.8 (9.1, 14.9)	12.23 (8.4, 16.93)	0.028
BUN mean, median (IQR)	18.33 (13.0, 29.4)	16.0 (12.0, 23.0)	25.5 (16.0, 44.0)	<0.001
Sodium mean, median (IQR)	138.0 (136.0, 140.2)	138.0 (136.0, 140.0)	138.0 (135.8, 140.5)	0.855
PT mean, median (IQR)	14.65 (13.6, 16.2)	14.35 (13.4, 15.55)	15.45 (14.1, 17.93)	<0.001
INR mean, median (IQR)	1.3 (1.2, 1.5)	1.27 (1.17, 1.4)	1.4 (1.25, 1.7)	<0.001
Potassium mean, median (IQR)	4.19 (3.87, 4.5)	4.15 (3.85, 4.45)	4.26 (3.9, 4.64)	<0.001
Platelet mean, median (IQR)	175.33 (130.33, 233.33)	188.67 (147.0, 245.5)	144.5 (100.5, 203.0)	<0.001
Lactate mean, median (IQR)	1.93 (1.4, 2.7)	1.8 (1.36, 2.43)	2.27 (1.6, 3.33)	<0.001
Hemoglobin mean, median (IQR)	10.15 (9.15, 11.4)	10.25 (9.23, 11.5)	9.95 (9.0, 11.1)	<0.001
PH mean, median (IQR)	7.38 (7.34, 7.41)	7.39 (7.35, 7.42)	7.35 (7.31, 7.4)	<0.001
Glucose mean, median (IQR)	130.57 (116.0, 150.89)	128.86 (115.67, 146.17)	134.57 (116.83, 158.67)	<0.001
Creatinine mean, median (IQR)	0.95 (0.72, 1.37)	0.85 (0.7, 1.1)	1.27 (0.88, 2.37)	<0.001
**Vital sign**				
SpO_2_ mean, median (IQR)	97.77 (96.52, 98.81)	97.88 (96.72, 98.92)	97.54 (96.18, 98.62)	<0.001
BT mean, median (IQR)	36.81 (36.43, 37.24)	36.82 (36.44, 37.21)	36.81 (36.4, 37.3)	0.975
Resp rate mean, median (IQR)	18.12 (16.08, 21.17)	17.71 (15.85, 20.36)	19.21 (16.68, 22.73)	<0.001
Mean bp mean, median (IQR)	75.11 (70.17, 81.36)	76.04 (71.1, 82.55)	73.55 (68.4, 78.7)	<0.001
Dias bp mean, median (IQR)	58.39 (53.15, 64.3)	59.07 (53.88, 65.26)	56.97 (51.87, 62.63)	<0.001
Sys bp mean, median (IQR)	112.86 (105.52, 122.26)	114.87 (107.21, 124.73)	109.52 (102.82, 118.0)	<0.001
Heartrate mean, median (IQR)	85.83 (77.53, 96.52)	84.6 (76.96, 94.64)	88.25 (79.15, 100.26)	<0.001
**Primary outcome**				
Death in hospital, *n* (%)	925 (15.109)	396 (9.880)	529 (25.024)	<0.001
Death 28-day, *n* (%)	1,114 (18.197)	484 (12.076)	630 (29.801)	<0.001
Death 90-day, *n* (%)	1,301 (21.251)	585 (14.596)	716 (33.869)	<0.001
Death 1-year, *n* (%)	1,606 (26.233)	779 (19.436)	827 (39.120)	<0.001
**Second outcome**				
AKI 7-day, *n* (%)	4,563 (74.534)	2,726 (68.014)	1,837 (86.897)	<0.001
Hospital stay ≥ 14 days, *n* (%)	2,092 (34.172)	1,171 (29.217)	921 (43.567)	<0.001
ICU stay ≥ 3 days, *n* (%)	3,422 (55.897)	1,935 (48.278)	1,487 (70.341)	<0.001
Survival time, median (IQR)	11.15 (6.26, 27.17)	10.04 (5.95, 23.28)	14.47 (7.45, 34.13)	<0.001

*BMI, body mass index; WBC, white blood cell count; BUN, Blood urea nitrogen; PT, prothrombin time; INR, international normalized ratio; SOFA, sequential organ failure assessment; SPASII, simplified acute physiology score; AKI, acute kidney injury; ICU, the intensive care unit; SPO2, pulse oxygen saturation; PH, potential of hydrogen; BT, body temperature; Data are represented as median (interquartile range) or n (%)*.

**Table 3:** The logistics regression of SOFA scoring system.

**Outcomes**	**Non-adjusted model**			**Adjusted model**		
	**OR**	**95% CL**	***p***	**OR**	**95% CL**	***p***
In-hospital death	3.044	2.64, 3.514	<0.001	2.86	2.471, 3.312	<0.001
28-day mortality	3.091	2.707, 3.532	<0.001	2.897	2.528, 3.323	<0.001
90-day mortality	2.997	2.643, 3.399	<0.001	2.817	2.475, 3.208	<0.001
One-year mortality	2.664	2.369, 2,995	<0.001	2.479	2.195, 2.8	<0.001
AKI 7-day	1.392	1.229, 1.578	<0.001	1.122	0.981, 1.285	0.095
Hospital stay ≥ 14 days day	1.87	1.676, 2.087	<0.001	1.744	1.557, 1.954	<0.001
ICU stay ≥ 3 days	2.541	2.272, 2.843	<0.001	2.444	2.18, 2.743	<0.001

*SOFA, sequential organ failure assessment; OR, odds ratio; CI, 95% confidence interval; AKI, acute kidney injury; ICU, intensive care unit; Adjusted for the confounders: resp rate mean, dias BP mean, sys BP mean, heart rate mean, bun mean, INR mean, potassium mean, lactate mean, hemoglobin mean, glucose mean, pH mean, SpO_2_ mean, BMI, age, renal failure, liver disease, hypertension, alcohol abuse, coagulopathy, congestive heart failure, gender*.

### Correlation Between SAPS II Scoring System and Postoperative Outcomes

The basic general data are summarized in Table 4 and deeply analyzed in Table 5. There were statistical differences between the groups of the SAPS II scoring system. The increased in-hospital mortality (OR 3.544, 95% CI 3.02, 4.164; *p* < 0.001), 28-day mortality (OR 3.92, 95% CI 3.376, 4.558; *p* < 0.001), 90-day mortality (OR 4.069, 95% CI 3.533, 4.693; *p* < 0.001) and 1-year mortality (OR 4.272, 95% CI 3.744, 4.879; *p* < 0.001) were obvious in the H-SAPS II group. The length of in-hospital stay (OR 2.912, 95% CI 2.581, 3.287; *p* < 0.001) and ICU stay (OR 2.997, 95% CI 2.651, 3.392; *p* < 0.001) was longer in the H-SAPS II group than the L-SAPS II group. The incidence of AKI within 7 days admitted to ICU was statistically significant (OR 1.464, 95% CI 1.258, 1.706; *p* < 0.001) in comparison.

**Table 4:** Basic characteristics of SAPS II scoring system.

**Characteristics**	**Overall (*n* = 6,122)**	**SPAS II <43 (*n* = 3,860)**	**SPAS II ≥ 43 (*n* = 2,262)**	***p***
SPASII, median (IQR)	38 (30, 48)	32.0 (27.0, 37.0)	52.0 (47.0, 60.0)	<0.001
Age, median (IQR)	66 (55, 76)	63.0 (53.0, 73.0)	71.0 (61.0, 79.0)	<0.001
Male gender, *n* (%)	3,704 (60.503)	2,398 (62.124)	1,306 (57.737)	<0.001
BMI, median (IQR)	27.84 (24.39, 31.84)	27.9 (24.42, 31.98)	27.77 (24.29, 31.63)	0.303
**Comorbidities**				
Drug abuse, *n* (%)	208 (3.398)	152 (3.938)	56 (2.476)	0.002
Alcohol abuse, *n* (%)	474 (7.743)	301 (7.798)	173 (7.648)	0.832
Coagulopathy, *n* (%)	1,153 (18.834)	545 (14.119)	608 (26.879)	<0.001
Liver disease, *n* (%)	463 (7.563)	244 (6.321)	219 (9.682)	<0.001
Renal failure, *n* (%)	869 (14.195)	357 (9.249)	512 (22.635)	<0.001
Hypothyroidism, *n* (%)	617 (10.078)	372 (9.637)	245 (10.831)	0.134
Diabetes, *n* (%)	1,448 (23.652)	883 (22.876)	565 (24.978)	0.062
Chronic pulmonary, *n* (%)	1,179 (19.258)	714 (18.497)	465 (20.557)	0.049
Hypertension, *n* (%)	758 (12.382)	326 (8.446)	432 (19.098)	<0.001
Congestive heart failure, *n* (%)	816 (13.329)	353 (9.145)	463 (20.469)	<0.001
**Laboratory test**				
WBC mean, median (IQR)	11.93 (8.9, 15.55)	11.7 (9.0, 14.77)	12.43 (8.72, 17.1)	<0.001
Bun mean, median (IQR)	18.33 (13.0, 29.4)	15.67 (12.0, 21.5)	27.5 (17.0, 44.33)	<0.001
Sodium mean, median (IQR)	138.0 (136.0, 140.2)	138.0 (136.2, 140.0)	138.0 (135.67, 140.71)	0.720
INR mean, median (IQR)	1.3 (1.2, 1.5)	1.3 (1.2, 1.4)	1.4 (1.2, 1.7)	<0.001
PT mean, median (IQR)	14.65 (13.6, 16.2)	14.42 (13.4, 15.65)	15.25 (13.85, 17.6)	<0.001
Potassium mean, median (IQR)	4.19 (3.87, 4.5)	4.15 (3.85, 4.44)	4.24 (3.9, 4.65)	<0.001
Platelet mean, median (IQR)	175.33 (130.33, 233.33)	179.33 (137.0, 233.0)	168.5 (117.67, 234.0)	<0.001
Lactate mean, median (IQR)	1.93 (1.4, 2.7)	1.88 (1.4, 2.5)	2.12 (1.5, 3.15)	<0.001
Hemoglobin mean, median (IQR)	10.15 (9.15, 11.4)	10.3 (9.25, 11.5)	9.92 (9.0, 11.1)	<0.001
Glucose mean, median (IQR)	130.57 (116.0, 150.89)	128.5 (115.33, 145.22)	134.75 (117.6, 160.33)	<0.001
Creatinine mean, median (IQR)	0.95 (0.72, 1.37)	0.87 (0.7, 1.1)	1.23 (0.85, 2.25)	<0.001
PH mean, median (IQR)	7.38 (7.34, 7.41)	7.38 (7.35, 7.42)	7.36 (7.31, 7.4)	<0.001
**Vital sign**				
SpO_2_ mean, median (IQR)	97.77 (96.52, 98.81)	97.82 (96.67, 98.82)	97.64 (96.28, 98.81)	<0.001
BT mean, median (IQR)	36.81 (36.43, 37.24)	36.84 (36.48, 37.24)	36.77 (36.35, 37.24)	<0.001
Res prate mean, median (IQR)	18.12 (16.08, 21.17)	17.72 (15.85, 20.41)	19.0 (16.57, 22.61)	<0.001
Mean bp mean, median (IQR)	75.11 (70.17, 81.36)	76.22 (71.22, 82.61)	73.4 (68.32, 78.79)	<0.001
Dias bp mean, median (IQR)	58.39 (53.15, 64.3)	59.56 (54.39, 65.59)	56.63 (51.14, 62.15)	<0.001
Sys bp mean, median (IQR)	112.86 (105.52, 122.26)	114.55 (107.05, 124.08)	110.26 (103.03, 119.36)	<0.001
Heart rate mean, median (IQR)	85.83 (77.53, 96.52)	84.89 (77.27, 94.66)	87.35 (78.06, 99.97)	<0.001
**Primary outcome**				
Death in-hospital, *n* (%)	925 (15.109)	293 (7.591)	632 (27.940)	<0.001
Death 28-day, *n* (%)	1,114 (18.197)	363 (9.404)	751 (33.201)	<0.001
Death 90-day, *n* (%)	1,301 (21.251)	446 (11.554)	855 (37.798)	<0.001
Death 1-year, *n* (%)	1,606 (26.233)	593 (15.363)	1,013 (44.783)	<0.001
**Second outcome**				
AKI 7-day, *n* (%)	4,563 (74.534)	2,621 (67.902)	1,942 (85.853)	<0.001
Hospital stay ≥ 14 days, *n* (%)	2,092 (34.172)	950 (24.611)	1,142 (50.486)	<0.001
ICU stay ≥ 3 days, *n* (%)	3,422 (55.897)	1,774 (45.959)	1,648 (72.856)	<0.001
Survival time, median (IQR)	11.15 (6.26, 27.17)	9.82 (5.86, 21.68)	15.3 (7.94, 41.25)	<0.001

*BMI, body mass index; WBC, white blood cell count; BUN, Blood urea nitrogen; PT, prothrombin time; INR, international normalized ratio; SOFA, sequential organ failure assessment; SPASII, simplified acute physiology score; AKI, acute kidney injury; ICU, the intensive care unit; SPO_2_, pulse oxygen saturation; PH,potential of hydrogen; BT,body temperature; Data are represented as median (interquartile range) or n (%)*.

**Table 5:** The logistics regression of SAPSII scoring system.

**Outcomes**	**Non-adjusted model**			**Adjusted model**		
	**OR**	**95% CL**	***p***	**OR**	**95% CL**	***p***
In-hospital death	3.839	3.318, 4.45	<0.001	3.544	3.02, 4.164	<0.001
28-day mortality	4.292	3.744, 4.928	<0.001	3.92	3.376, 4.558	<0.001
90-day mortality	4.471	3.929, 5.093	<0.001	4.069	3.533, 4.693	<0.001
One-year mortality	4.748	4.208, 5.362	<0.001	4.272	3.744, 4.879	<0.001
AKI 7-day	2.869	2.502, 3.291	<0.001	1.464	1.258, 1.706	<0.001
Hospital stay ≥ 14 days	3.123	2.798, 3.488	<0.001	2.912	2.581, 3.287	<0.001
ICU stay ≥ 3 days	3.156	2.822, 3.533	<0.001	2.997	2.651,3.392	<0.001

*SPASII, simplified acute physiology score; OR, odds ratio; CI, 95% confidence interval; AKI, acute kidney injury; ICU: the intensive care unit. Adjusted for the confounders: PT mean, INR mean, platelet mean, lactate mean, hemoglobin mean, glucose mean, creatinine mean, PH mean, SpO_2_ mean, resp rate mean, mean BP mean, dias bp mean, BMI, congestive heart failure, drug abuse, coagulopathy, liver disease, renal failure, hypertension, gender*.

### Comparison of Decision Curves

**Figure 4:**
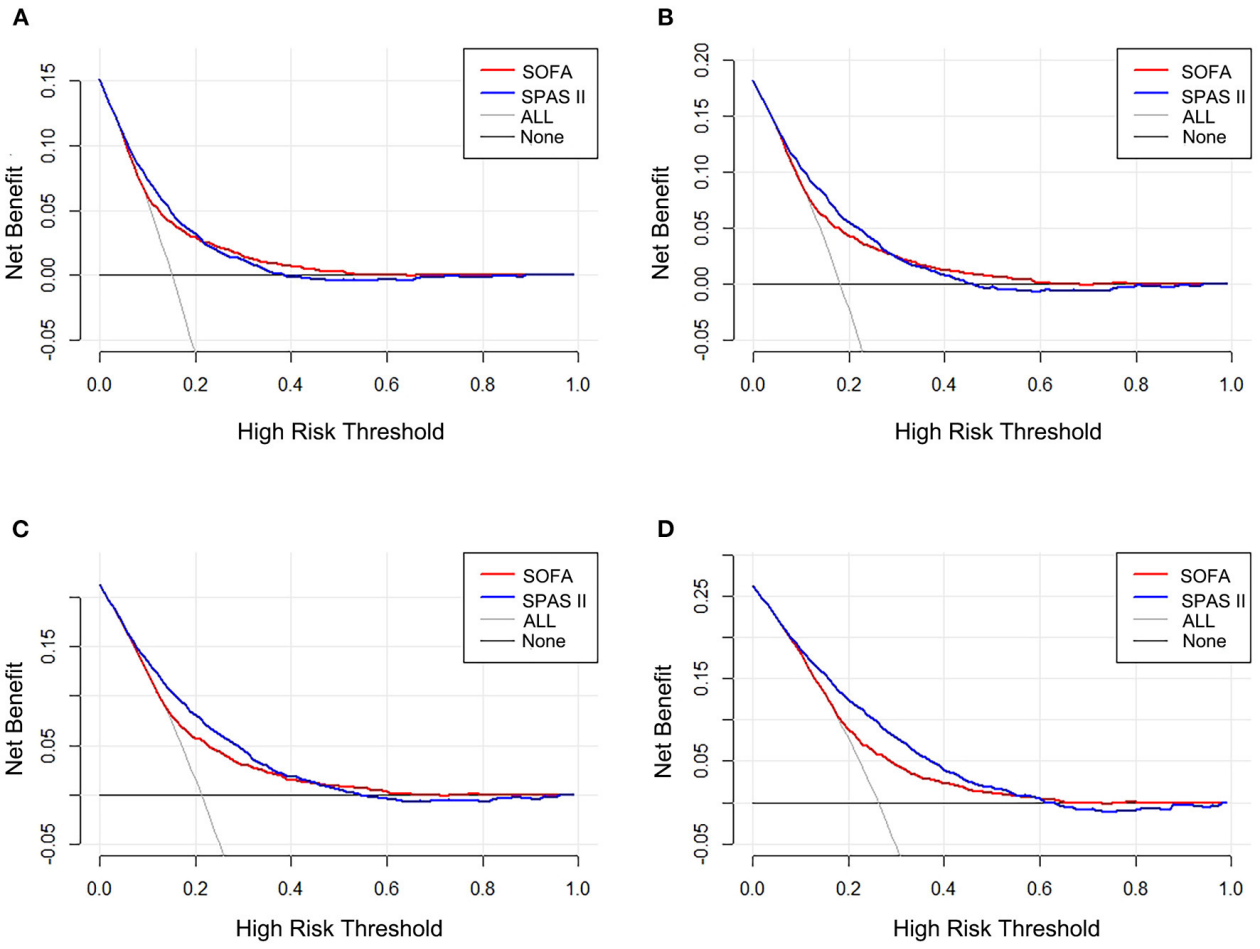
Decision curve analysis (DCA) of the two scoring systems. **(A)** In-hospital mortality, **(B)** 28-day mortality, **(C)** 90-day mortality, and **(D)** 1-year mortality.

As exhibited in Figure 4, the DCA curve of the SAPS II scoring system was higher than that of the SOFA scoring system in predicting 1-year mortality and 90-day mortality. Otherwise, the net benefit was not different between the two scoring systems in in-hospital mortality and 28-day mortality. This suggests that the SAPS-II scoring system is more meaningful than SOFA in assessing long-term mortality.

## Discussion

This retrospective study demonstrated that a score of 7 in the SOFA scoring system and a score of 43 in the SAPS II scoring system with the first 24 h after ICU admission were warning values for predicting the risk of outcomes. Exceeding the warning values of the SOFA score and SAPS II score was associated with elevated mortality, prolonged ICU interval, and hospital interval. Besides, the incidence of AKI was increased in the SAPS II scoring system but not in the SOFA scoring system.

After cardiac surgery, patients have the risk of organ dysfunction or even deterioration, which predicted a poorer prognosis. The aim of the SOFA score was to objectively and quantitatively evaluate the severity of six organ systems dysfunction over time. It consisted of respiratory, circulatory, renal, hematology, hepatic, and central nervous systems, which are related to the recovery of patients with cardiac surgery (17). Although its main role is to predict organ dysfunction, the association between quantification of SOFA score and survival was inevitable (18). A previous systematic review showed that the SOFA model based on 24 h after ICU admission could be used to predict mortality (19). Other types of patients were also effectively evaluated by SOFA score, including post-cardiac arrest syndrome (20), people requiring extracorporeal cardiopulmonary resuscitation (21), critically ill cirrhotic patients with acute decompensation (22), patients with acute respiratory failure in intensive care unit (23), contemporary cardiac intensive care unit population (24), critically ill elderly patients with acute infective endocarditis (25), extracorporeal membrane oxygenation (ECMO)-treated acute myocardial infarction (AMI) patients (26)—some of above studies behaved even well. In the cardiac surgery population, the previous literature stated that SOFA score had good discriminative power for hospital mortality (27); the same observation was also confirmed in Ceriani et al.'s research (28). Doerr et al.'s study had found that SOFA score could predict 30-day mortality (29). In our study, we used a larger sample size to test the predictive effect of SOFA scores on short-term and long-term mortality. Results are remarkable on the performance of SOFA score as previous studies, especially above the cutoff value. The warning value of the SOFA scoring system is able to assess the risk of outcomes as a new valuable observation index in post-cardiac patients.

The SAPS II scoring system consisted of physiological variables, basic characteristics, and several complications, which could provide an estimate of the risk of death on the basis of large samples with the independence of primary diagnosis, and also was developed for the evaluation of the efficiency of ICUs (30). Recent research established that the SAPS II score could predict the prognosis and in-hospital mortality in AMI patients treated with ECMO with a good performance (26). But it could not ser good predictor for discharged mortality in small sample size studies (31). The result of the previous study of more than 2,000 patients after cardiac surgery showed that the SAPS II score had good discrimination in-hospital mortality (27). The same role was confirmed in another post-cardiac surgery population (32). These researches were consistent with part of our findings. Moreover, previous literature had claimed that the SAPS II scoring system could be implemented reliably while mortality was closely related to the rater's scoring practice (33). This can be interpreted as the diversities of results. Although overestimates of mortality were reported by some researchers (34–37), the objective of our study was for early warning based on the threshold of the SAPS II scoring system.

Decision curve analysis is a method widely used to evaluate the clinical utility of specific models (38). The curve with the highest benefit score at a given threshold was determined to be the best choice (16). A recent study involving multiple scoring systems confirmed the superiority of the SAPS II scoring system in predicting mortality through DCA among sepsis patients (39). Besides, Abraham Schoe et al.'s research including more than 3,600 post-cardiac surgery patients has demonstrated that SOFA score and SAPS II score could predict hospital mortality, while SAPS II was better (32). This tendency is similar to our finding: The SAPS II scoring system had more net benefits on assessing the long-term mortality compared with the SOFA scoring system. As a comprehensive scoring system for postoperative multiple organ physiological function during post-cardiac surgery patients, the SAPS II scoring system may perform better in clinical application.

As for the complexity of progressing in disease, it is hard to find a perfect score of predicting risk comprehensively. The strength of this study lies in identifying warning values of the SOFA score and SAPS II score and giving new insight into the reference value of the SOFA and SAPS II scoring system. Moreover, it enriched the methods of early detecting the prognosis in patients with cardiac surgery and might be used as decision support for clinical intervention. The scoring system was tested in the discrimination of long-term mortality in a large sample of patients with cardiac surgery. However, it was a retrospective observational study which raised possible bias caused by heterogeneous factors. This study focuses on the whole group after cardiac surgery, but we believe that the changes in organ function after different cardiac surgery can also be reflected through the scoring system. Of course, it is also a perfect direction to further explore the differences in the scoring system in different types of cardiac surgery. Moreover, the collected data were from over 10 years ago, these conclusions may not be feasible nowadays with the improvement of surgical technology and ICU treatment level. In this large sample retrospective analysis, the short-term results are consistent with existing studies. There are still few longer-term results. These results can provide clinicians with a warning SOFA and SAPS II value, but the specific implementation should be treated with caution. More retrospective and prospective clinical studies are necessary for verification in the future.

## Conclusion

This study suggested that exceeding the cutoff values of the SOFA score and SAPS II score could lead to increased mortality, prolonged length of ICU stay, and in-hospital stay. Score 7 in the SOFA scoring system and score 43 in the SAPS II scoring system with the first 24 h after ICU admission were warning values for worse outcomes. The SAPS II scoring system had a better discriminative performance of 90-day mortality and 1-year mortality in post-cardiac surgery patients than the SOFA scoring system. Focusing on the critical value of the scoring system is of significance for treatment in ICU.

## Data Availability Statement

The datasets presented in this study can be found in online repositories. The names of the repository/repositories and accession numbers can be found below: https://mimic.mit.edu/docs/iii/tables/.

## Ethics Statement

Access to the database was reviewed and approved by the Institutional Review Committee of Beth Israel Deaconess Medical Center and Beth Israel deacons Medical Center (Approval Code 10323541). Written informed consent was not required for this study, in accordance with the local legislation and institutional requirements.

## Author Contributions

FX gathered and processed the data. FX, WL, and CZ prepared the results. FX, WL, and CZ contributed to writing the manuscript. FX and RC put forward the idea. RC revised the manuscript. All the authors read and approved the final manuscript.

## Funding

This project is supported by the Sichuan Science and Technology Program, China (Grant No. 2019YFS0352).

## Conflict of Interest

The authors declare that the research was conducted in the absence of any commercial or financial relationships that could be construed as a potential conflict of interest.

## Publisher's Note

All claims expressed in this article are solely those of the authors and do not necessarily represent those of their affiliated organizations, or those of the publisher, the editors and the reviewers. Any product that may be evaluated in this article, or claim that may be made by its manufacturer, is not guaranteed or endorsed by the publisher.
